# Patients with a Wide Range of Disorders Related to *WFS1* Gene Variants: Novel Mutations and Genotype–Phenotype Correlations

**DOI:** 10.3390/genes15121592

**Published:** 2024-12-12

**Authors:** Julia Grzybowska-Adamowicz, Karolina Gadzalska, Paulina Jakiel, Ewa Juścińska, Monika Gorządek, Sebastian Skoczylas, Tomasz Płoszaj, Przemysława Jarosz-Chobot, Irina Kowalska, Małgorzata Myśliwiec, Agnieszka Szadkowska, Agnieszka Zmysłowska

**Affiliations:** 1 Department of Clinical Genetics, Medical University of Lodz, Pomorska Str. 251, 92-213 Lodz, Poland; 2 Department of Children’s Diabetolog and Lifestyle Medicine, Medical University of Silesia, 40-055 Katowice, Poland; 3 Department of Endocrinology, Diabetology and Internal Medicine, Medical University of Bialystok, 15-089 Bialystok, Poland; 4 Department of Pediatrics, Diabetology and Endocrinology, Medical University of Gdansk, 80-211 Gdansk, Poland; 5 Department of Pediatrics, Diabetology, Endocrinology and Nephrology, Medical University of Lodz, 92-213 Lodz, Poland

**Keywords:** *WFS1*-spectrum disorders, Wolfram syndrome, Wolfram-like syndrome, genotype–phenotype correlation

## Abstract

**Background:** *WFS1*-spectrum disorders are caused by a mutation in the *WFS1* gene. The term includes a wide range of rare disorders, from the most severe Wolfram syndrome with autosomal recessive inheritance to milder clinical manifestations with a single causative variant in the *WFS1* gene, such as Wolfram-like syndrome, low-frequency sensorineural hearing loss (LFSNHL), isolated diabetes mellitus (DM), nonsyndromic optic atrophy (OA), and isolated congenital cataracts. **Methods:** The aim of this study was to evaluate genotype–phenotype correlations in Polish patients with *WFS1*-spectrum disorders. The study group constituted 22 patients (10 F; 12 M), including 10 patients (3 F; 7 M) referred to the Outpatient Clinic for Rare Diseases in Children and Adolescents and Diabetogenetics between 2019 and 2024 with clinical symptoms suggestive of *WFS1*-spectrum disorders, and 12 of their first-degree relatives (7 F; 5 M) from 10 families in Poland. Molecular testing was performed using tNGS (*Targeted Next Generation Sequencing*; Illumina) and analyzed for variants in the *WFS1* gene. **Results**: Thirteen different variants in the *WFS1* gene were found in 22 individuals (10 patients and family members), including the identification of two new variants (c.1535T>C and c.2485C>G). All patients had hyperglycemia or DM, hearing impairment, OA, or a combination of these symptoms. Four patients in the study group were diagnosed with Wolfram syndrome and all were compound heterozygotes for variants in the *WFS1* gene. **Conclusions:** The evaluation of molecular characteristics in combination with clinical symptoms broadens the understanding of *WFS1*-spectrum disorders and allows more accurate management and prognosis for patients with this diagnosis.

## 1. Introduction

*WFS1*-spectrum disorder (*WFS1*-SD) is caused by a mutation in the *WFS1* gene, ranging from the phenotypically most severe Wolfram syndrome to diseases with a milder clinical course. Wolfram syndrome (WS) is a rare neurodegenerative disorder with an autosomal recessive way of inheritance and an estimated prevalence of 1 in 770,000 [1]. The main clinical manifestations correspond to the acronym DIDMOAD, which includes diabetes insipidus (DI), diabetes mellitus (DM), optic atrophy (OA), and deafness (D). The syndrome usually starts in childhood with insulin-dependent diabetes mellitus and optic atrophy. Other complications such as urological, neurological, and psychiatric problems are also observed [1,2]. Markers of the clinical course of WS and effective causal treatments are still being identified [3,4,5,6].

*WFS1*-SD also contains various disorders caused by heterozygous variants in the *WFS1* gene, which are described as nonclassic *WFS1*-SD. The term includes a broad range of diseases with milder phenotype than patients with WS, sharing some of its clinical features, mainly ophthalmologic abnormalities, diabetes mellitus, or hearing impairment. The group consists of Wolfram like syndrome (WFS-like syndrome), low-frequency sensorineural hearing loss (LFSNHL), isolated diabetes mellitus, nonsyndromic optic atrophy, and isolated congenital cataracts. The prevalence of these conditions is yet to be known [5,7].

The complexity and variability in clinical manifestations caused by mutations in the *WFS1* gene prompted a search for indicators of the development of particular disorders from this heterogeneous spectrum. The genotype–phenotype correlations were already studied in WS [8,9,10]. Some recent studies were focused on evaluation whether defined types of variants found in the *WFS1* gene might be responsible for specific clinical phenotypes [5,8,9]. Researchers have proposed different approaches in terms of segregating variants into categories. Some have defined the genotypic classes based on the protein product [8], whereas others have used classification based on mutation type [5,9]. De Heredia et al. described that patients with expression of a defective WFS1 protein had earlier onset of the characteristic symptoms [8]. Rigoli et al. and Lee et al. used slightly different assignments of mutation types to groups. However, the common conclusion was that patients with nonsense and frameshift variants present more severe clinical features [5,9]. Moreover, genotype–phenotype correlations were recently described in WFS-like syndrome [7]. The study described that missense variants in the *WFS1* gene lead to a milder disease course. They were also associated with earlier development of hearing impairment. Patients with loss-of-function mutation were described to have earlier age of onset of diabetes mellitus, optic atrophy, and were more likely to develop neurological symptoms [7]. Recently, the coexistence of optic atrophy and hearing loss was described to be common phenotype in patients with heterozygous *WFS1* variants [7,11]. So far, the strict correlations between specific variants and presented phenotype have not been described. Thus, clinical manifestations remain fundamental in assigning the right diagnosis to the patient [12]. Recent consensus emphasizes the importance of multidisciplinary evaluation and the use of next-generation sequencing (NGS) in the diagnosis of WS [13].

The aim of this study was to analyze the causative variants of the *WFS1* gene found in Polish patients with *WFS1*-spectrum disorders and to search for genotype–phenotype correlations.

## 2. Patients and Methods

### 2.1. Patients

The study group consisted of 22 patients (10 F, 12 M), including 10 patients (3 F, 7 M; mean age at diagnosis 21.1 ± 16.3 years) referred to the Rare Disease Outpatient Clinic for Children and Adolescents and Diabetogenetics in 2019–2024 with clinical symptoms suggesting WFS1-spectrum disorders and 12 of their first-degree relatives (7 F, 5 M; mean age 39.6 ± 17.2 years) from 10 families in Poland. All patients fulfilled the diagnostic criteria for WFS1-SD spectrum disorder. Patients had hyperglycemia or diabetes mellitus diagnosed based on WHO criteria (*n* = 3), coexisting hyperglycemia or DM with hearing loss based on audiometry (*n* = 3), coexisting hyperglycemia or DM and optic atrophy confirmed by OCT (optical coherence tomography) (*n* = 2), or coexisting glucose tolerance disorders and OA and hearing loss (*n* = 2). Clinical assessment revealed additional signs and symptoms associated with WFS1-SD (Table 1). Moreover, the daughter of one patient had a neurogenic bladder and two more participants presented with neurological disorders. All patients and/or their relatives expressed written consent to participate in the study.

For direct sequencing of the *WFS1* gene, DNA was extracted from blood drawn in EDTA containers using standard procedures.

### 2.2. Sequencing

#### 2.2.1. Library Preparation

DNA was extracted from peripheral blood using a Maxwell^®^ RSC Instrument (Maxwell^®^ RSC Blood DNA Kit, Promega, Madison, WI, USA). The library was prepared using the Agilent SureSelect QXT Target Enrichment protocol with a custom gene panel, in accordance with the manufacturer’s instructions. The paired-end sequencing was performed on a NextSeq550 System, Illumina (2 × 150 bp).

#### 2.2.2. Bioinformatics Analysis

Bioinformatic analysis was conducted by mapping the FASTQ files to the GRCh38/hg38 reference genome using the BWA-MEM algorithm [14], and then duplicates were removed using Picard tools. Bcftools was applied to VCF files manipulation. For variant calling we used DeepVariant algorithm [15] and variant annotation was performed using VEP (Variant Effect Predictor) [16].

#### 2.2.3. Variants Selection Criteria

The variants of the *WFS1* gene were filtered in the GnomAD v4.1 database for the European population, with a frequency below 0.01 [17]. Variants were then filtered for their damaging potential using REVEL [18] and CADD [19]. The American College of Medical Genetics and Genomics (ACMG) classification was also applied [20].

#### 2.2.4. Variant Confirmation

Segregation in families and confirmation of variants in probands was performed by Sanger sequencing on a 3500 Series Genetic Analyzer (Applied Biosystems, Waltham, MA, USA). Sequence analysis was performed using BLAST: Basic Local Alignment Search Tool (National Institutes of Health) [21].

## 3. Results

Genotyping of 10 patients revealed 13 different variants in the *WFS1* gene, including 2 novel mutations, 11 of them were pathogenic/likely pathogenic and 2 were variants of unknown significance (VUS). One VUS—c.1535T>C—not reported so far, with low frequency in the general population and highly conserved, appeared to be segregated in the family and associated with clinical symptoms. Subsequently, the c.68C>T variant was initially probably benign, but its classification was changed to VUS due to segregation of the variant in the family. In addition, one pathogenic c.2485C>G variant was found to be a spontaneous, de novo variant.

Interestingly, one patient (#6.1) was found to have two coexisting monogenic diabetes. Molecular tests identified a pathogenic variant in the *GCK* gene, confirming GCK-MODY (maturity onset diabetes of young) apart from WFS-like syndrome (variant c.68C>T).

All the identified variants are summarized in Table 2. Molecular analysis of first-degree relatives confirmed previously found variants in 12 individuals. All analyzed relatives were heterozygous.

In family #1, a 12-year-old boy was referred with suspected Wolfram syndrome. At 4 years of age, the patient experienced polydipsia, polyuria, weight loss and was diagnosed with diabetes. He was treated with insulin therapy. At the age of 6, the patient’s visual acuity decreased. Bilateral optic atrophy was confirmed in OCT examination. Hearing test results were normal. Molecular tests identified two causative variants in the *WFS1* gene (c.932G>A and c.1698_1703del). The mother and father of the patient were found to have heterozygous variant in the gene. Unfortunately, they were unavailable for further examination to evaluate the symptoms of *WFS1*-SD.

In family #2, a 16-year-old male was diagnosed with hyperglycemia; impaired fasting glycaemia (IFG) was recognized in oral glucose tolerance test (OGTT). Additionally, the patient had hypothyroidism and astigmatism. His sister and father were also diagnosed with IFG. Molecular analysis identified the presence of a single likely pathogenic variant (c.2054G>A) of the *WFS1* gene in all three members of the family. They were all diagnosed with isolated hyperglycemia.

In family #3, a 4-year-old girl experienced polydipsia, polyuria, and slight loss of body mass. The patient was diagnosed with diabetes mellitus (glycated hemoglobin; HbA1c: 11.7%, random blood glucose 433 mg/dL, low fasting C-peptide, negative antibodies characteristic of type 1 diabetes). She was treated with insulin therapy. In addition, the patient had hearing impairment. She was referred with suspected monogenic diabetes. The genetic testing identified the presence of two heterozygous variants in the *WFS1* gene (c.1619G>A and c.1535T>C). The patient was diagnosed with Wolfram syndrome. An analysis of variants within the family revealed that both parents had heterozygous variant in the *WFS1* gene. The mother had hearing impairment confirmed by audiometry and bilateral optic nerve atrophy in the upper and lower quadrant of the retinal nerve fiber layer (RNFL) in OCT study. Her father had had diabetes mellitus for 3 years and was diagnosed with isolated diabetes mellitus.

In family #4, an 18-year-old male was suspected to have monogenic diabetes because of episodes of hyperglycemia since 14 years of age. He also had cochlear implants due to congenital bilateral hearing loss. In the OGTT test, both IFG and impaired glucose tolerance (IGT) were recognized. HbA1c was 5.4% and islet-related antibodies were negative. He was treated with metformin. Short stature was also noted due to somatotropin deficiency. However, growth hormone therapy was not administered because of glucose intolerance. Atrioventricular tachycardia was also present. In the OCT examination, no abnormalities were found. The patient’s mother had hearing impairment and atrioventricular tachycardia, but no glycemic disturbances were noted. The patient had confirmed WFS-like syndrome and his mother was diagnosed with LFSNHL caused by likely pathogenic *WFS1* gene (c.467C>T).

In family #5, a 20-year-old male was suspected to have Wolfram syndrome. The patient had congenital bilateral hearing loss and diabetes mellitus. He experienced loss of visual acuity at 16 years of age. Ophthalmological evaluation revealed partial optic atrophy. He also had behavioural problems. Genetic testing identified two causative mutations in the *WFS1* gene (c.1079G>A and c.2051C>T). His father had hearing loss and diabetes mellitus. Analysis of variants within the family revealed that both parents had heterozygous variant in the *WFS1* gene. The patient was diagnosed with Wolfram syndrome and the father with WFS-like syndrome. The variant (c.1079G>A) was present in the mother, who experienced hearing loss.

**Figure 1:**
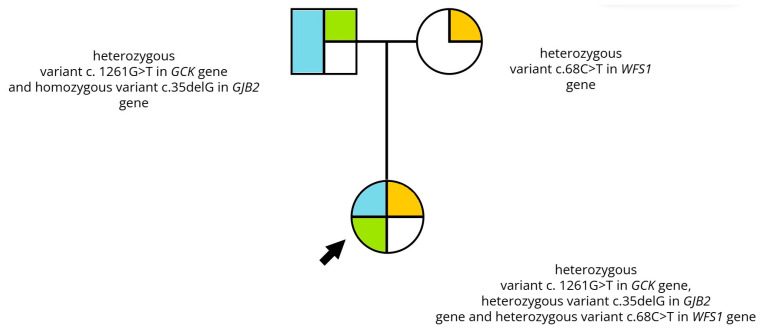
Pedigree of family #6 with two coexisting monogenic diabetes. Mutation in *GJB2* gene (blue), mutation in *GCK* gene (green), mutation in *WFS1* gene (orange).

In family #6, a 32-year-old female was referred due to suspected MODY diabetes. Diabetes mellitus with uncharacteristic symptoms was identified by OGTT at 14 years of age. Autoantibodies characteristic for type 1 diabetes were negative. The patient was treated with diet alone, and then, due to inadequate glycemic control, a sulfonylurea derivative was added. In OCT examination, no abnormalities were found. Audiometry revealed a slight hearing loss. Additionally, the patient had anxiety and vertigo. Molecular tests found a pathogenic variant in *GCK* gene, confirming GCK-MODY diabetes and additionally a heterozygous pathogenic variant in the *GJB2* gene that identified the patient as a carrier of nonsyndromic hearing loss, and a heterozygous variant in the *WFS1* gene (c.68C>T). Analysis of variants within the family revealed the father to have a homozygous pathogenic variant in the *GJB2* gene, which clarified the cause of his hearing loss, and a heterozygous variant in the *GCK* gene, responsible for GCK-MODY diabetes. In addition, the variant in the *WFS1* gene (c.68C>T) was found in the patient’s mother. She had hearing impairment and hemiparesis due to an ischemic attack 10 years prior. The patient was diagnosed with WSF-like syndrome (Figure 1).

In family #7, a 10-year-old boy was referred with suspected Wolfram syndrome. He had congenital bilateral hearing loss. At 5 years of age, he was diagnosed with short stature caused by somatotropin hypopituitarism. During growth hormone therapy, diabetes was diagnosed by OGTT test and insulin therapy was started. Then, OCT examination confirmed optic atrophy. Genetic testing revealed a single pathogenic variant in the *WFS1* gene (c.2051C>T). Mutation segregation analysis in the family showed no variants in the parents. The patient with WFS-like syndrome was described in the detail earlier by our team [22].

In family #8, a young female was referred with insulin-dependent diabetes mellitus identified at 13 years of age. Next, she experienced severe loss of visual acuity. Genetic testing identified two heterozygous variants in the *WFS1* gene (c.1672C>T and c.1234_1237delGTCT), and she was diagnosed with Wolfram syndrome. Later, her 5-year-old daughter was also referred to the Department. The daughter was born with hydrocephalus and spina bifida in the thoracolumbar region. She also had neurogenic bladder. In the molecular tests, a single pathogenic variant in the *WFS1* gene was found. Currently, she does not present symptoms typical for *WFS1*-SD.

In family #9, a 62-year-old man was referred with suspected monogenic diabetes. At age 57, the patient was diagnosed with diabetes by OGTT. In addition, HbA1c was 8.3%, no insulin secretion and negative antibodies characteristic for type 1 diabetes were found. He was treated with metformin and insulin. He was also diagnosed with myopia. In his family history, both parents had diabetes, his daughter had hyperglycemia, and his father had schizophrenia. According to a single likely pathogenic variant in the *WFS1* gene found in molecular testing (c.2149G>A), isolated diabetes mellitus was confirmed.

In family #10, an 11-year-old boy was suspected to have monogenic diabetes. At 8 years of age, the patient was diagnosed with diabetes mellitus. The patient had decreased insulin secretion and negative islet-related antibodies and he was still treated with insulin therapy. In OCT examination and audiometry no abnormalities were found. Molecular analysis identified the presence of one pathogenic variant (c.1619G>A) and one VUS in the *WFS1* gene (c.2485C>G). Analysis of variants in the family revealed a heterozygous pathogenic variant in the *WFS1* gene in the patient’s father, who has isolated hyperglycemia, with no other disorders. The mother had none of the variants. Subsequent reclassification of the variant in bioinformatics databases recognized the variant as pathogenic (c.2485C>G). The patient was initially diagnosed with isolated diabetes. However, we were unable to provide follow-up information regarding whether the patient developed other clinical manifestations in the course of *WFS1*-SD.

**Figure 2:**
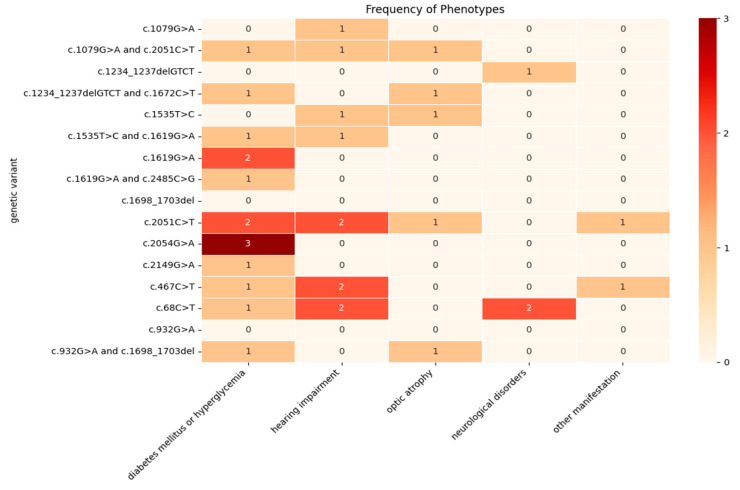
Genotype–phenotype correlations between *WFS1* gene variants and clinical manifestations.

Figure 2 shows genotype–phenotype correlations relating the occurrence of various clinical symptoms to specific variants in our patients. Glucose tolerance disorders described as diabetes or hyperglycemia showed an association with most of the variants, but mainly with the c.2054G>A, 2051C>T and 1619G>A variants. A strong association was also observed between hearing impairment and the c.68C>T, c.467 C>T and c.2051C>T variants (Figure 2).

## 4. Discussion

Among all patients, four were diagnosed with Wolfram syndrome, three with WFS-like syndrome, and three with isolated diabetes or hyperglycemia. Among the patient’s relatives, one was diagnosed with WFS-like syndrome, four with isolated diabetes or hyperglycemia, and one with LFSNL. In addition, a 7-year-old girl currently does not present symptoms characteristic for *WFS1*-SD apart from neurogenic bladder, but she may develop them in the future. Unfortunately, the lack of detailed clinical data made a definitive diagnosis impossible in five relatives. Patients diagnosed with WS were compound heterozygous in terms of mutations in the *WFS1* gene and all of them were treated with insulin therapy. Other patients and all first-degree relatives were found to have heterozygous variants in the *WFS1* gene. Furthermore, we were able to correlate the variants found with clinical symptoms in patients and family members, which indicated that the c.2054G>A, 2051C>T and 1619G>A variants are strongly associated with impaired glucose tolerance. Patients with the c.2054G>A variant followed a reduced glycemic index diet. Patients with variants 2051C>T and 1619G>A were treated with insulin therapy or metformin before their genetic diagnosis. We also observed the relationship of the c.68C>T, c.467C>T and c.2051C>T variants with hearing impairment.

In recent years, attempts have been made to assess genotype–phenotype correlations in terms of mutation types rather than specific variants [7,8]. Most of the *WFS1* variants found in our current study were missense mutations, in contrast to our earlier work [23], where most of the identified variants were nonsense or frameshift mutations. However, that study included patients with confirmed Wolfram syndrome and their family members, whereas in our current study, most patients had a milder phenotype. According to the results presented by de Muijnck et al., patients with missense variants in the *WFS1* gene had fewer clinical symptoms and a lower risk of developing diabetes insipidus, which is in agreement with our observations. de Muijnck et al. also noted that patients with missense variants had a younger age at onset of hearing impairment (median 1.5 years) [7]. Very early onset of hearing loss occurred in some of our patients (#4.1, #5.1 and #7.1). However, other patients developed hearing impairment later in life or were not previously diagnosed with hearing impairment.

Among ten mutations found in our study, eight of them have been previously described in the literature. Seven of those variants (c.1698_1703del, c.1619G>A, c.467C>T, c.2051C>T, c.1234_1237delGTCT, c.2149G>A, and c.1672C>T) were described in the review on genotype–phenotype correlation regarding WS [8]. Three variants (c.2051C>T, c.1234_1237delGTCT and c.1619G>A) were categorized as genotype mutation type III associated with genotype class C by Heredia et al. [8]. This class was associated with earlier onset of the characteristic symptoms as well as urological complications. In our study, there were three patients diagnosed with WS, who had at least one of the mentioned variants (c.2051C>T, c.1234_1237delGTCT and c.1619G>A). Two of them (#8.1 and #3.1) were diagnosed with diabetes before 16 years of age, whereas one patient (#5.1) also had hearing impairment, optic atrophy, and behavioral problems. Patient #8.1 with the c.1234_1237delGTCT variant was diagnosed with diabetes mellitus and optic atrophy, which corresponds to earlier report [24]. The comparison of the phenotype of patients with heterozygous variants is difficult to make as the review was focused on WS and not on the milder diseases from *WFS1*-SD. Three patients (#3.3, #10.1 and #10.2) with c.1619G>A had isolated diabetes or hyperglycemia, and two patients (#5.2 and #7.1) with c.2051C>T were diagnosed with WFS-like syndrome.

The c.2051C>T variant was also found in patients with coexisting hearing impairment and optic atrophy with heterozygous variant in the *WFS1* gene [25] and in a patient with Wolfram syndrome as one of two missense mutations [26]. de Muijnck et al. stated that the c.2051C>T variant was the most commonly reported *WFS1* variant in WFS-like syndrome studies [7]. Additionally, the phenotype of patients with the heterozygous c.2051C>T variant comprised hearing impairment and optic atrophy with less frequently developed diabetes mellitus, which corresponds with the clinical characteristic of the patient (#7.1) and quite strong connection between hearing impairment and this variant. described in our study. Moreover, recently, a knock-in *Wfs1^E864K^* line mouse was generated, which will provide new opportunities for researchers [27]. Most recently, de Muijnck et al. presented a study on optic atrophy associated with autosomal dominant *WFS1*-SD, where 10 patients with c.2051C>T variant were described. All of them were found to have optic atrophy and hearing impairment, whereas only three had diabetes mellitus. Additionally, three patients experienced growth abnormalities and two had mental health problems [11], which is in accordance with our results, as growth hormone deficiency was diagnosed in patient #7.1 and behavioral problems were noted in family #5.

Furthermore, one of the variants (c.2054G>A) was mentioned in a study on early-onset diabetes in a Chinese population [28], where a patient with diabetes with this variant also had another pathogenic variant in the *WFS1* gene. In our study, members of family #2 were identified to have a single c.2054G>A variant and presented hyperglycemia.

Noteworthily, two of the variants we found (c.1535T>C and c.2485C>G) are new, not described to date. The patient (#3.1) with the c.1535T>C variant had a second variant, which has already been reported. Her mother (#3.2) had optic atrophy and hearing impairment. The c.2485C>G variant was found in a patient (#10.1) who also had a second, known variant in the *WFS1* gene. The patient had diabetes mellitus without abnormalities on OCT or audiometric examination. Unfortunately, further clinical information was not available. The variant posed a diagnostic challenge because it was not segregated in the family. It is worth mentioning that the c.2485C>G (p.Leu829Val) variant was identified at an identical amino acid position to the variant documented previously in the ClinVar database—c.2486T>C (p.Leu829Pro) (accession: VCV000004521.15) and was classified as pathogenic. The in silico tool predicts a pathogenic effect for this variant. The c.2486T>C variant has been previously described in patients with hearing impairment and in two sisters who also had another variant, with hearing loss and optic atrophy [29,30].

A limitation of the study is the relatively small number of participants studied, as *WFS1*-SD are very rare diseases [1,2]. Next, the analysis in the study is retrospective, which limits valuable information from longer observation periods and follow-up visits. In addition, in some patients, especially their family members, we were unable to obtain information on visual or hearing impairment in the absence of OCT or audiometry testing. Moreover, the changing classification of variants over time provides a diagnostic challenge.

In conclusion, our study focused on describing phenotype–genotype correlations in patients with disorders caused by variants in the *WFS1* gene. The study provided insight into the impact of specific variants on patients with both Wolfram syndrome and milder disorders in the form of *WFS1*-SD. In addition, we identified two new variants in the *WFS1* gene, which expands the spectrum of variants identified in this gene. Evaluating the variants and their molecular characteristics in comparison with clinical manifestations enables more precise treatment and prognosis for future patients diagnosed with *WFS1*-SD.

## Figures and Tables

**Table 1:** Clinical symptoms in patients with *WFS*1-SDs and their first-degree relatives.

Family. Patient Number	Sex (F/M)	Age at Genetic Diagnosis (Years)	Patient (P)/ Family Member (FM)	Final Diagnosis	Variant 1	Variant 2	DM or Hyperglycemia (Age of Onset—Years)	Hearing Impairment (Age of Onset—Years)	Optic Atrophy (Age of Onset—Years)	Neurological Disorders (Age of Onset—Years)	Other Manifestation	DM/Hyperglycemia Treatment—(Insulin Dose in Units/kg of Body Weight)
#1.1	M	12	P	WS	c.932G>A	c.1698_1703del	Yes (4)	No	Yes (6)	No		Insulin therapy (0.74 U/kg)
#1.2	F	38	FM	NA	c.1698_1703del		No	No	No	No		NA
#1.3	M	39	FM	NA	c.932G>A		No	No	No	No		NA
#2.1	M	19	P	Isolated hyperglycemia	c.2054G>A		Yes (16)	No	No	No		Diet
#2.2	M	43	FM	Isolated hyperglycemia	c.2054G>A		Yes (39)	No	No	No		Diet
#2.3	F	14	FM	Isolated hyperglycemia	c.2054G>A		Yes (13)	No	No	No		Diet
#3.1	F	6	P	WS	c.1535T>C	c.1619G>A	Yes (4)	Yes (6)	No	No		Insulin therapy (0.47 U/kg)
#3.2	F	29	FM	Hearing impairment and optic atrophy	c.1535T>C		No	Yes (1)	Yes (29)	No		NA
#3.3	M	37	FM	Isolated diabetes mellitus	c.1619G>A		Yes (34)	No	No	No		Metformin
#4.1	M	19	P	WFS-like syndrome	c.467C>T		Yes (14)	Yes (0,1)	No	No	Short stature	Metformin
#4.2	F	54	FM	LFSNHL	c.467C>T		No	Yes	No	No		NA
#5.1	M	20	P	WS	c.1079G>A	c.2051C>T	Yes (20)	Yes (0,1)	Yes (16)	No		Insulin therapy (0.71 U/kg)
#5.2	M	52	FM	WFS-like syndrome	c.2051C>T		Yes (46)	Yes (0,1)	No	No		NA
#5.3	F	51	FM	Hearing loss	c.1079G>A		No	Yes	No	No		NA
#6.1 *	F	33	P	WFS-like syndrome	c.68C>T		Yes (14)	Yes (33)	No	Yes (29)		Sulfonylurea derivative
#6.2	F	67	FM	Hearing impairment	c.68C>T		No	Yes (0,1)	No	Yes (57)		NA
#7.1	M	9	P	WFS-like syndrome	c.2051C>T		Yes (5)	Yes (0,1)	Yes (7)	No	Short stature	Insulin therapy (0.8 U/kg)
#8.1	F	20	FM	WS	c.1234_1237delGTCT	c.1672C>T	Yes (13)	No	Yes (19)	No		Insulin therapy (0.62 U/kg)
#8.2	F	5	P	Currently without symptoms typical for *WFS1*-SD	c.1234_1237delGTCT		No	No	No	Yes (3)		NA
#9.1	M	62	P	Isolated diabetes mellitus	c.2149G>A		Yes (57)	No	No	No		Metformin and insulin therapy (0.43 U/kg)
#10.1	M	11	P	Isolated diabetes mellitus	c.1619G>A	c.2485C>G	Yes (8)	No	No	No		Insulin therapy (0.91 U/kg)
#10.2	M	46	FM	Isolated hyperglycemia	c.1619G>A		Yes	No	No	No		Diet

* The patient also has GCK-MODY diabetes; NA—not applicable; DM—diabetes mellitus; WS—Wolfram syndrome; *WFS1*-SD—*WFS1* spectrum disorders; LFSNHL—low-frequency sensorineural hearing loss; U/kg—units/kilograms of body weight.

**Table 2:** Classification of variants in the *WFS1* gene found among patients and their first-degree relatives.

Nucleotide Change *	Amino Acid Change	ACMG	Clinvar	gnomAD 4.1 European (Non-Finnish) Allele Count/Allele Number	Type of Mutation
c.1079G>A	p.Cys360Tyr	Likely Pathogenic	VUS 6x	251/1112008	Missense
c.1234_1237delGTCT	p.Val412SerfsTer29	Pathogenic	Pathogenic 2x	3/1111958	Frameshift, nonsense
**c.1535T>C**	**p.Leu512Pro**	**VUS**	**NA**	**0/1111938**	**Missense**
c.1619G>A	p.Trp540Ter	Pathogenic	Pathogenic 1x; Likely Pathogenic 1x	6/1111996	Nonsense
c.1672C>T	p.Arg558Cys	Pathogenic	Pathogenic 6x; Likely Pathogenic 6x; VUS 3x	89/1111992	Missense
c.1698_1703del	p.Leu567_Phe568del	Pathogenic	Pathogenic 2x; Likely Pathogenic 4x	28/1111870	Deletion
c.2051C>T	p.Ala684Val	Pathogenic	Pathogenic 17x; Likely Pathogenic 2x	3/1111910	Missense
c.2054G>A	p.Arg685His	Likely Pathogenic	Likely Pathogenic 1x; VUS x6; Likely Benign 1x	208/1111898	Missense
c.2149G>A	p.Glu717Lys	Pathogenic	Likely Pathogenic 2x	38/1111898	Missense
**c.2485C>G**	**p.Leu829Val**	**Pathogenic**	**NA**	**5/1106662**	**Missense**
c.467C>T	p.Thr156Met	Likely Pathogenic	VUS 1x	5/1111994	Missense
c.68C>T	p.Ala23Val	VUS	VUS 2x	24/1098202	Missense
c.932G>A	p.Gly311Asp	Likely Pathogenic	VUS 1x	NA	Missense

* GenBank accession number NM_006005.3; ACMG—American College of Medical Genetics and Genomics classification, NA—not applicable, VUS—variant of unknown significance. The new variants are bolded.

## Data Availability

The data may be made available to all interested persons upon request to the correspondent author.
